# Comparative Transcriptome Analysis of *Penicillium citrinum* Cultured with Different Carbon Sources Identifies Genes Involved in Citrinin Biosynthesis

**DOI:** 10.3390/toxins9020069

**Published:** 2017-02-21

**Authors:** Taotao Li, Guoxiang Jiang, Hongxia Qu, Yong Wang, Yehui Xiong, Qijie Jian, Yu Wu, Xuewu Duan, Xiangrong Zhu, Wenzhong Hu, Jiasheng Wang, Liang Gong, Yueming Jiang

**Affiliations:** 1 Key Laboratory of Plant Resource Conservation and Sustainable Utilization, Guangdong Provincial Key Laboratory of Applied Botany, South China Botanical Garden, Chinese Academy of Sciences, Guangzhou 510650, China; wubangcai88@163.com (T.L.); 13145206@163.com (G.J.); q-hxia@scbg.ac.cn (H.Q.); qijiejan@163.com (Q.J.); 18255137100@163.com (Y.W.); xwduan@scib.ac.cn (X.D.); xiangrongchu@163.com (X.Z.); 2 Zhong Shan Entry-Exit Inspection and Quarantine Bureau, Zhongshan 528403, China; wrone@163.com; 3 Innovation Center for Structural Biology, Tsinghua-Peking Joint Center for Life Sciences, School of Life Sciences, Tsinghua University, Beijing 100084, China; yehuixiong@mail.tsinghua.edu.cn; 4 College of life science, University of Chinese Academy of Sciences, Beijing 100039, China; 5 Department of Food Engineering, College of Life Science, Dalian Nationalities University, Dalian 116600, China; hwz@dlnu.edu.cn; 6 Department of Environmental Health Science, University of Georgia, Athens, GA 30602, USA; jswang@uga.edu

**Keywords:** citrinin, glucose, *Penicillium citrinum*, polyketide biosynthesis, sucrose

## Abstract

Citrinin is a toxic secondary metabolite of *Penicillium citrinum* and its contamination in many food items has been widely reported. However, research on the citrinin biosynthesis pathway and its regulation mechanism in *P. citrinum* is rarely reported. In this study, we investigated the effect of different carbon sources on citrinin production by *P. citrinum* and used transcriptome analysis to study the underlying molecular mechanism. Our results indicated that glucose, used as the sole carbon source, could significantly promote citrinin production by *P. citrinum* in Czapek’s broth medium compared with sucrose. A total of 19,967 unigenes were annotated by BLAST in Nr, Nt, Swiss-Prot and Kyoto Encyclopedia of Genes and Genomes (KEGG) databases. Transcriptome comparison between *P. citrinum* cultured with sucrose and glucose revealed 1085 differentially expressed unigenes. Among them, 610 were upregulated while 475 were downregulated under glucose as compared to sucrose. KEGG pathway and Gene ontology (GO) analysis indicated that many metabolic processes (e.g., carbohydrate, secondary metabolism, fatty acid and amino acid metabolism) were affected, and potentially interesting genes that encoded putative components of signal transduction, stress response and transcription factor were identified. These genes obviously had important impacts on their regulation in citrinin biosynthesis, which provides a better understanding of the molecular mechanism of citrinin biosynthesis by *P. citrinum*.

## 1. Introduction

Mycotoxins are secondary fungal metabolites, and are toxic to humans and animals [1]. Mycotoxins contamination in agricultural products has been of great global concern during the past several decades. Citrinin is a mycotoxin that was originally isolated from *Penicillium citrinum* (*P. citrinum*), and was also produced by several other species of *Penicillium*, *Aspergillus*, and *Monascus* [2,3]. *P. citrinum* can be found in different environments, ranging from agricultural fields, permafrost sediments to forest soils [4], which have been vigorously studied since the discovery of their capability to produce citrinin [5]. Contamination of *P. citrinum* and citrinin has been reported in a wide variety of agricultural commodities, food and feedstuffs [6]. Additionally, citrinin was also found in many fruits [7,8]. Toxicologically, citrinin is hepatotoxic and nephrotoxic to a number of animal species and has been implicated in endemic animal and human nephropathy [9,10].

Citrinin is synthesized via the polyketide pathway from acetate, malonate and methionine to make a trimethylated pentaketide. This is then acted on by various oxidoreductase to create citrinin [11,12]. Ten enzymatic steps and related gene clusters for citrinin biosynthesis were widely investigated [12,13,14]. The inhibition of citrinin biosynthesis was also previously reported. For example, the citrinin production can be inhibited by fatty acids or their corresponding methylketones [15]. Hajjaj et al. (2011) reported that citrinin production could be drastically reduced or even eliminated by some amino acids in *Monascus ruber* [16]. Tan et al. (2014) provided proteomic insight into the effect of ethanol on the citrinin biosynthesis pathway in *Monascus purpureus* [17]. However, researches related to *P. citrinum* and citrinin mainly focused on developing methods to remove citrinin from food and feed [18,19,20] or the influence of different culture conditions on citrinin accumulation [2,21]. Hence, research on the citrinin biosynthesis pathway and its regulation mechanism in *P. citrinum* is rarely found. Furthermore, carbon sources including sucrose and glucose have been well known for their different impacts on mycotoxin production such as trichothecene [22], patulin [23], ochratoxin [24], and fumonisin B2 [25]. Additionally, sucrose and glucose are two main sugars included in the values of total soluble solids in fruits, and the relationship between sucrose concentration and patulin production was investigated in *P. expansum* [26]. Barad et al. (2014) also showed that different sugars induce the accumulation of patulin with different efficiencies, with sucrose being the most efficient inducer for patulin accumulation in *P. expansum* comparing with glucose and gluconic acid [27]. In contrast, Ruiz et al. (2010) suggested that glucose and sucrose can repress fungal secondary metabolism through carbon catabolite repression [28]. Although these previous studies provide useful information to elucidate the role of carbon sources in mycotoxin production, different fungi species might have different responses to carbon sources due to their genetic variability and the complexity of mycotoxin biosynthesis pathways [29]. Therefore, it is necessary to clarify the regulation mechanism of citrinin biosynthesis by *P. citrinum* cultured with different carbon sources.

Citrinin biosynthesis is quite complex and study of its process by focusing on single gene/protein functions remains difficult. In recent years, high-throughput RNA-Seq has attracted increasing interest as an approach to investigate the gene expression profile in filamentous fungi. Indeed, transcriptome analysis using RNA-Seq technology has already been applied to several filamentous fungi for investigation of the mycotoxin biosynthesis and infection mechanism [30,31,32]. To the best of our knowledge, there is no information available about the transcriptome analysis of citrinin production by *P. citrinum*.

In this study, we investigated the impact of different carbon sources (sucrose and glucose) on citrinin production by *P. citrinum.* Differentially expressed genes (DEGs) were analyzed using transcriptome sequencing to identify key genes involved in citrinin biosynthesis and transcriptional regulation. Further, a possible gene regulatory network for the citrinin production in *P. citrinum* is proposed. New information about the citrinin biosynthesis pathway and the regulation mechanism is provided, which might be useful for devolving a strategy for early prevention of citrinin contamination.

## 2. Results

### 2.1. Effect of Different Carbon Sources on Citrinin Production by P. citrinum and Intracellular H_2_O_2_ Content

**Figure 1:**
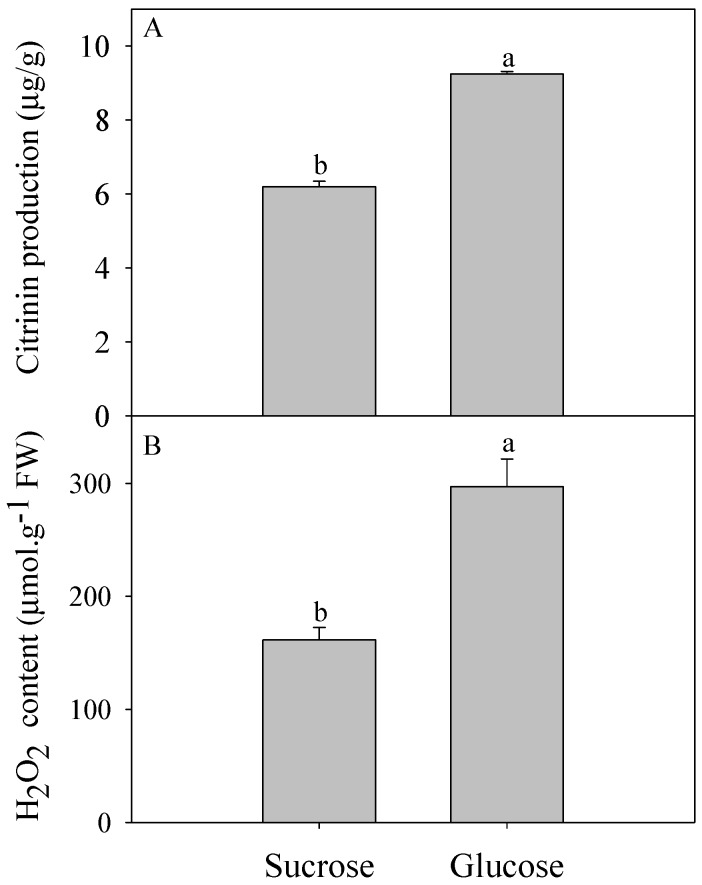
Citrinin production (**A**) and H_2_O_2_ content (**B**) of *P. citrinum* cultured with different carbon sources. Different letters represent significant differences (*p* < 0.05).

Figure 1 shows the citrinin production by *P. citrinum* and H_2_O_2_ content after a 7-day culture in medium with either sucrose or glucose as the sole carbon source. Results indicated that *P. citrinum* could produce citrinin in culture medium with either of the two carbon sources. However, citrinin produced by glucose-cultured *P. citrinum* (9.25 μg/g) was significantly higher than that by sucrose-cultured *P. citrinum* (6.19 μg/g) (*p* < 0.05, Figure 1A). To confirm whether different carbon sources lead to changes in metabolism of reactive oxygen species (ROS) in *P. citrinum*, intracellular H_2_O_2_ content was determined in 7-day-old mycelia from two kinds of culture broths. Again, glucose-cultured *P. citrinum* significantly increased in intracellular H_2_O_2_ content as compared with that of sucrose-cultured *P. citrinum* (*p* < 0.01, Figure 1B).

### 2.2. Sequencing, Assembly and Annotation

High-throughput RNA sequencing was performed for each mycelia sample with Q20 greater than 95.2% and less than 0.02% low quality reads (Supplementary Materials Table S1). Assembly of the reads generated a total of 25,491 unigenes, with an N50 of 3889 and average length of 2048 bp (Supplementary Materials Table S2).

Among the 25,491 unigenes, 19,967 unigenes could be annotated by BLAST in the four databases including Nr, Nt, Swiss-Prot and KEGG (Kyoto Encyclopedia of Genes and Genomes), whereas the remaining 5524 unigenes could not be annotated in these databases. Specifically, 19,588, 13,114, 13,934 and 13,921 unigenes were annotated in Nr, Nt, Swiss-Prot and KEGG databases (Supplementary Materials Figure S1), respectively. From the annotation in the Nr database, the proportion of genes with E-values ranging from 1 × 10^−5^ to 1 × 10^−45^ was 30.3%, and the proportion of genes with E-values less than 1 × 10^−45^ was 69.7%, suggesting that strong sequence homology was identified (Supplementary Materials Figure S2A). Species distribution indicated that 46.3% unigenes of *P. citrinum* were the best blasts matches to *P. brasilianum*, and 4.6% unigenes were matched to *P. camemberti* (Supplementary Materials Figure S2B).

To gain insight into functions of these annotated genes, Gene Ontology (GO) and Clusters of Orthologous Groups of proteins (COG) functional classification were further performed and their results were shown in Supplementary Materials Figures S3 and S4. The spearman correlation coefficient for sucrose-cultured samples (S1 and S2), and glucose-cultured samples (G1 and G2) were greater than 0.91, suggesting the consistency between the biological replicates (Supplementary Materials Figure S5).

### 2.3. Analysis of DEGs between Sucrose- and Glucose-Cultured P. citrinum

Comparative transcriptome analysis of sucrose- and glucose-cultured *P. citrinum* mycelia was conducted, which was based on the combined transcriptome assembly of all these samples. A total of 1085 differentially expressed genes (DEGs) were identified. Among them, 475 genes were down-regulated and 610 genes were up-regulated in glucose-cultured sample as compared with those in sucrose-cultured sample.

Gene function analysis was performed to obtain more information from the large quantities of data. GO functional classification for the 1085 DEGs was made using Blast2GO software. In the domain of biological process, DEGs were distributed among 17 categories (Supplementary Materials Figure S6), the largest one was “metabolic process” (465 genes), followed by “cellular process” (394 genes), “single-organism process” (368 genes) and “localization” (137 genes). In the domain of molecular function, the largest category was “catalytic activity” (385 genes), followed by “binding” (280 genes). In addition, there were many genes that were classified as “transporter activity” genes (63) and “nucleic acid binding transcription factor activity” genes (30). In the domain of cellular component, DEGs were classified into “cell” (237 genes), “cell part” (236 genes), “organelle” (173 genes), “membrane” (150 genes), “membrane part” (125 genes), “organelle part” (71 genes), “macromolecular complex” (69 genes), “membrane-enclosed lumen” (29), “extracellular region” (22 genes). To investigate the biological functions of these DEGs, GO enrichment analysis was performed. There were 13 significantly enriched GO terms in the biological process category (Table 1). GO term (GO: 0055114) related to the oxidation-reduction process had the highest number of genes, followed by GO term (GO: 0071705) related to nitrogen compound transport.

KEGG pathway analysis classified the 1085 DEGs into 21 categories, of which the largest one was ‘Metabolic pathways’ (255 genes), followed by ‘Carbohydrate metabolism’ (168 genes), ‘Amino acid metabolism’ (156 genes) and “Biosynthesis of secondary metabolites” (123 genes) (Supplementary Materials Figure S7). These classifications suggest that these genes may play pivotal roles in primary and secondary metabolism during citrinin biosynthesis. To further understand how carbon source regulates citrinin biosynthesis at the molecular level in *P. citrinum*, the DEGs were mapped to the terms in the KEGG database. By comparing with the whole genome background, we identified significantly enriched metabolic pathways or signal transduction pathways. A total of six pathways including “Biosynthesis of secondary metabolites”, “Glycine, serine and threonine metabolism”, “Steroid biosynthesis”, “Fatty acid biosynthesis”, etc., were significantly enriched (Q value < 0.05) (Table 2).

### 2.4. DEGs Involved in Secondary Metabolism in Sucrose- and Glucose-Cultured P. citrinum

Of the genes involved in secondary metabolism, 123 genes were differentially expressed in *P. citrinum* cultured with different carbon sources. Among them, 81 genes were up-regulated and 42 genes were down-regulated in the glucose-cultured *P. citrinum* as compared with that of the sucrose-cultured. These genes were involved in metabolisms of polyketide, steroid, cell wall, fatty acid and phenylalanine, and shikimate pathway, etc., (Supplementary Materials Excel S1). It was also found that *Alcohol dehydrogenase* (CL348.Contig2_All, CL3211.Contig2_All and Unigene4605_All) and *Aldehyde dehydrogenase* (Unigene5443_All) were up-regulated in the glucose-cultured *P. citrinum*. Interestingly, most genes related to cytochrome P450s were up-regulated in the glucose-cultured *P. citrinum* as compared with those in sucrose-cultured *P. citrinum* (Supplementary Materials Excel S1).

**Figure 2:**
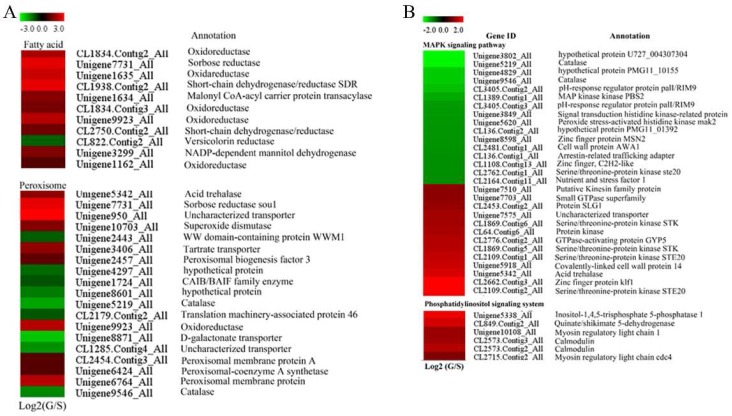

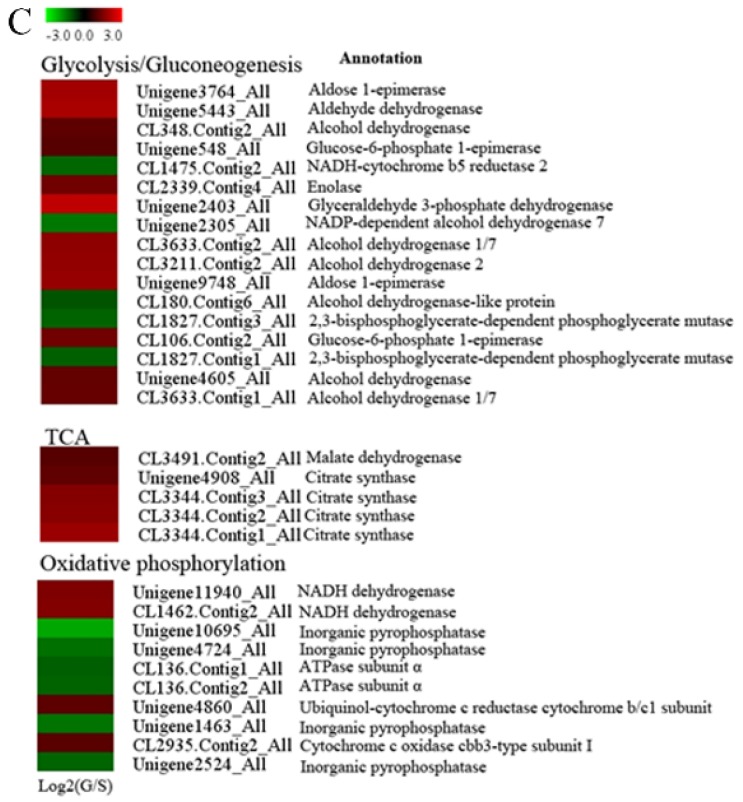
Expression profiles of DEGs involved in fatty acid biosynthesis, peroxisome metabolism (**A**); signaling pathway (**B**); and primary metabolism (**C**).

Steroid biosynthesis was also found in the significantly enriched metabolic pathways in the KEGG database. Among the genes involved in steroid biosynthesis, only four of them were down-regulated in the glucose-cultured *P. citrinum*, while 13 of them were up-regulated including *cytochrome P450 61* (Unigene4470_All), *transcriptional regulatory protein pro-1* (Unigene1201_All) and *lipase* (CL3490.Contig2_All). Ten genes classified as fatty acid biosynthesis genes were up-regulated in the glucose-cultured *P. citrinum,* while only one fatty acid biosynthesis related gene was down-regulated (Figure 2A). Most of them were annotated as *oxidoreductase*.

### 2.5. Peroxisome- and Proteasome-Related Genes

Peroxisome-related genes showed different expression patterns between sucrose- and glucose-cultured *P. citrinum*. Our results indicated that 19 peroxisome-related genes were significant DEGs (Figure 2A), 10 were up-regulated while nine were down-regulated in glucose-cultured *P. citrinum*. It is worth noting that the *superoxide dismutase* gene (Unigene10703_All) showed up-regulation while the *catalase* gene (Unigene5219_All and Unigene9546_All) showed down-regulation in the glucose-cultured *P. citrinum* as compared with those of sucrose-cultured *P. citrinum*.

Four genes were identified as proteasome-related genes such as *26S proteasome regulatory subunit* (CL2096.Contig1_All and CL1365.Contig2_All), *proteasome subunit beta type-3* (Unigene4489_All) and *proteasome subunit beta type-4* (Unigene6626_All). All of these genes were up-regulated in the glucose-cultured *P. citrinum*, which indicated that glucose might result in protein degradation in *P. citrinum*.

### 2.6. Genes Involved in Signal Transduction

The functions of 35 signal transduction genes were analyzed using KEGG pathway analysis. The results suggested that citrinin biosynthesis might be regulated coordinately by various factors, including a phosphatidylinositol signaling system and the mitogen-activated protein kinase (MAPK) signaling pathway (Figure 2B). In the MAPK signaling pathway, five serine/threonine-protein kinases were detected. Glucose-cultured *P. citrinum* showed higher expression of four of them (CL1869.Contig6_All, CL1869.Contig5_All, CL2109.Contig1_All and CL2109.Contig2_All), but lower expression of the remaining one than that of sucrose-cultured *P. citrinum* (Figure 2B). In addition, two *GTPase* genes (Unigene7703_All and CL2776.Contig2_All) and one *protein kinase* gene (CL64.Contig6_All) were also up-regulated in glucose-cultured *P. citrinum*, but two histidine kinase mak2 (Unigene3849_All and Unigene5620_All) were down-regulated. Interestingly, all the genes involved in phosphatidylinositol signaling were significantly up-regulated in glucose-cultured *P. citrinum* (Figure 2B). Furthermore, we also found that the *Serine/threonine-protein kinase*, *GTPase* and *histidine kinase mak2* genes were involved in response to the stimulus process (Supplementary Materials Excel S1).

### 2.7. Transcription Factors Genes

Genes encoding transcription factors, including Zn(II)2Cys6 transcription factor, GATA transcription factor (AreA), were detected, which were also DEGs between glucose- and sucrose-cultured *P. citrinum*. All the differentially expressed Zn(II)2Cys6 transcription factors were up-regulated, while GATA transcription factors were down-regulated in the glucose-cultured *P. citrinum* as compared with those in the sucrose-cultured (Supplementary Materials Figure S8). It is worth noting that the GATA transcriptional activator (*areA*) was also found in the response to stimulus process (Supplementary Materials Excel S1). Besides *areA* and Zn(II)2Cys6 transcription factors, qRT-PCR confirmation showed that *LaeA* was up-regulated while *pacC* was just slightly up-regulated in the glucose-cultured *P. citrinum* as compared with those in the sucrose-cultured *P. citrinum* (Supplementary Materials Figure S9).

### 2.8. Genes Involved in Primary Metabolism

DEGs associated with primary metabolism were mainly involved in glycolysis, citrate cycle, amino acid metabolism, fructose and mannose metabolism, etc. Among them, genes involved in glycolysis, including *enolase* (CL2339.Contig4_All), *glucose-6-phosphate 1-epimerase* (Unigene548_All) and *glyceraldehyde-3-phosphate dehydrogenase* (Unigene2403_All), were up-regulated in the glucose-cultured *P. citrinum* as compared with those in the sucrose-cultured. Interestingly, all genes involved in the tricarboxylic acid (TCA) cycle were also up-regulated in the glucose-cultured *P. citrinum* (Figure 2C). In addition, genes involved in energy metabolism also showed different expression patterns between glucose- and sucrose-cultured *P. citrinum*. Specifically, genes involved in the electron transport chain (ETC), such as *NADH dehydrogenase* (Unigene11940_All and CL1462.Contig2_All), *ubiquinol-cytochrome c reductase* cytochrome b/c1 subunit (Unigene4860_All) and *cytochrome c oxidase cbb3-type subunit I* (CL2935.Contig2_All), were all up-regulated, while genes related to ATP synthesis including *ATPase subunit α* (CL136.Contig1_All and CL136.Contig2_All) and *inorganic pyrophosphatase* (Unigene1463_All and Unigene2524_All) were down-regulated in the glucose-cultured *P. citrinum* as compared with those in the sucrose-cultured.

Amino acid metabolism of *P. citrinum* was significantly altered by different carbon sources in this study. We identified 156 genes involved in amino acid metabolism, including glutamate, alanine, proline, valine, leucine and histidine and so on (Supplementary Materials Figure S10). Among 15 genes involved in histidine metabolism, 13 genes were up-regulated in the glucose-cultured *P. citrinum* as compared with those in the sucrose-cultured. In addition, most of genes involved in alanine, aspartate, glutamate, leucine and valine metabolism were also up-regulated in the glucose-cultured *P. citrinum*, except for CL2945.Contig2_All (*carbamoyl-phosphate synthase*) and CL2563.Contig1_All (*3-ketoacyl-CoA thiolase A*). However, most genes involved in proline metabolism were down-regulated in the glucose-cultured *P. citrinum* (Supplementary Materials Figure S10).

### 2.9. Quantitative Real-Time PCR (qRT-PCR) Confirmation

**Figure 3:**
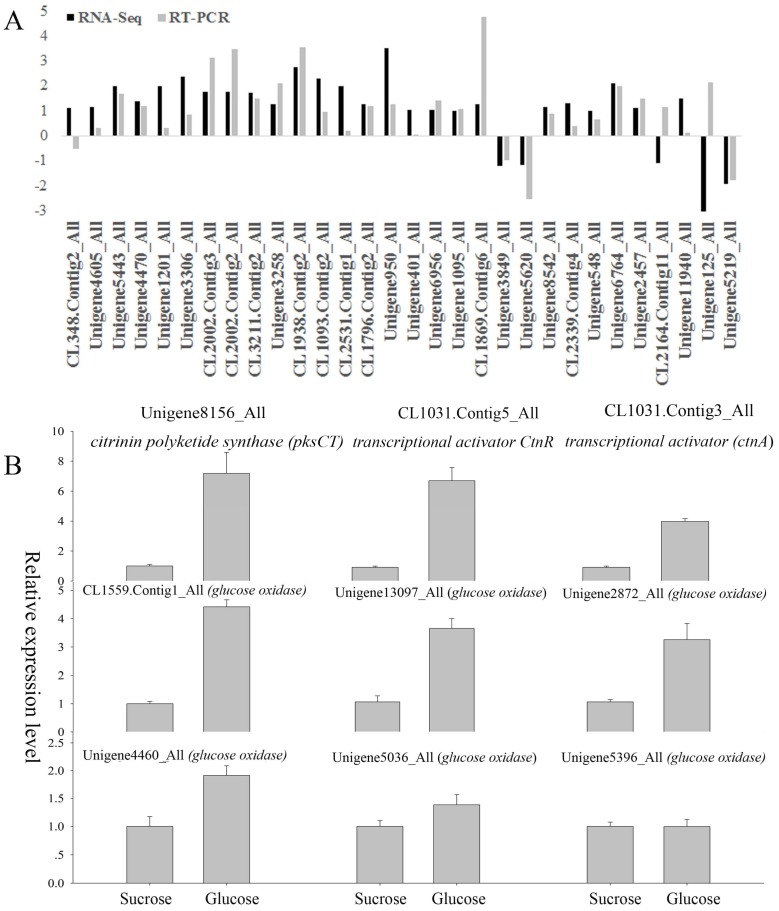
Conformation of the RNA-seq data by quantitative real-time PCR (**A**); and analysis of the expression levels of key genes in the citrinin biosynthesis pathways reported for other fungi, *glucose oxidase* genes (**B**).

Quantitative real-time PCR (qRT-PCR) was used to confirm findings on the RNA-seq data. Thirty genes were selected according to the following criteria: (a) genes had significantly differential expression between sucrose- and glucose-cultured samples; and (b) genes were previously reported to be potentially involved in citrinin biosynthesis. Results of the qRT-PCR analysis generally confirmed what was found in the RNA-seq data (Figure 3A). The expression patterns of 27 genes by RNA-seq were the same as those by the qRT-PCR approach, and only three genes (CL348.Contig2_All, CL2164.Contig11_All and Unigene125_All) showed different expression patterns between these two approaches. Additionally, we also selected key cDNAs in citrinin biosynthesis pathways reported for other fungi and quantified their transcript levels by qRT-PCR. From the transcriptome data, it was found that Unigene8156_All was annotated as *Monascus ruber* strain M7 *citrinin polyketide synthase* (*pksCT*), CL1031.Contig5_All as *citrinin biosynthesis transcriptional activator* *ctnR* [*Monascus purpureus*], and CL1031.Contig3_All as *Monascus ruber* strain M7 *citrinin biosynthesis transcriptional activator* (*ctnA*). According to qRT-PCR results, all of these genes were up-regulated in the glucose-cultured *P. citrinum* as compared with those in the sucrose-cultured *P. citrinum* (Figure 3B). In order to investigate the source of H_2_O_2_ and whether glucose oxidase is involved in citrinin production, the expression level of six genes annotated as glucose oxidase in our transcriptome data were further analyzed. Five of them were up-regulated in the glucose-cultured *P. citrinum* as compared with those in the sucrose-cultured *P. citrinum*; only one (Unigene 5396_All) was not differentially expressed under the two carbon sources (Figure 3B).

## 3. Discussions

Considering the importance of sugar in fruits described by Kumar et al. [26], we investigated the effect of different carbon sources (sucrose and glucose) on citrinin production by *P. citrinum* and used transcriptome analysis to study the underlying mechanism at the molecular level. This study will expand our knowledge of the genetic regulation of citrinin biosynthesis in *P. citrinum*.

### 3.1. Oxidative Stress Involved in Citrinin Biosynthesis by P. citrinum

Oxidative stress is associated with the biosynthesis of secondary metabolites including mycotoxins, which was well reviewed previously [33]. ROS metabolism in the fungal cells was reported to affect the production of trichothecene and ochratoxin A [34,35]. A recent study investigated the regulation of citrinin production in relation to oxidative stresses in *P. verrucosum* and found that production of citrinin was induced under oxidative stresses [36]. Mitochondria is a major source of ROS, which are produced by incomplete reduction of oxygen with electrons that have leaked from respiratory chain complexes [37]. Previous research showed that inhibition of mitochondrial ATP synthesis could result in a significant increase in O^2−^ production [38]. In this study, genes involved in ATP synthesis were down-regulated in the glucose-cultured *P. citrinum* (Figure 2C), indicating that the fungi cells were not in great demand of ATP in the presence of glucose. In addition, ETC genes were all up-regulated under glucose condition, resulting in a high Δp (protonmotive force) that could favor ROS production in mitochondria [38]. In sum, the differential expression of these genes in *P. citrinum* cultured with different carbon sources might result in an imbalance of oxidative phosphorylation and ROS metabolism. The up-regulation of proteasome-related genes also confirmed that more ubiquitin-labeled proteins were transferred to the proteasome in response to ROS stress.

Peroxisomes are also involved in the catabolism of ROS, and metabolism of oxygen free radicals in peroxisomes has important effects on cellular metabolism [39,40]. Our results showed that the genes involved in peroxisome metabolism (peroxisomal membrane protein, peroxisomal-coenzyme A synthetase and peroxisomal biogenesis factor 3) were up-regulated in the glucose-cultured *P. citrinum*. In addition, the up-regulation of superoxide dismutase (SOD) and down-regulation of catalase (CAT) in peroxisome (Figure 2A) induced higher accumulation of H_2_O_2_ under the glucose condition than under the sucrose condition (Figure 1B), which imposed oxidative stress on the *P. citrinum*. It is worth noting that glucose itself is also involved with H_2_O_2_ production. Glucose oxidase (GOX) catalyzed the oxidation of d-glucose to d-gluconolactone and hydrogen peroxide [41]. Therefore, the oxidation of glucose would be another important source of H_2_O_2_. In this study, except for Unigene 5396_All, five *glucose oxidase* genes were up-regulated in the glucose-cultured *P. citrinum* as compared with those in the sucrose-cultured *P. citrinum* (Figure 3B). These data indicate that glucose oxidase may also be involved in the citrinin biosynthesis via regulating the ROS production in *P. citrinum*. Taken together, the oxidative stress in *P. citrinum* caused by glucose can induce the citrinin biosynthesis.

### 3.2. DEGs Involved in Secondary Metabolism

It is difficult to study the regulation of the polyketide biosynthesis pathway in *P. citrinum* because of the nature of its complexity. Polyketides are a broad class of secondary metabolites synthesized by microbial organisms [14] and citrinin was the first compound identified as a polyketide [12]. The polyketide pathway is the major route for formation of secondary metabolites including citrinin, which was synthesized from acetyl-CoA and malonyl-CoA (with molar ration of 1:4) by PKS3 [14]. Our results indicated that the glucose-cultured *P. citrinum* showed higher expressions of genes involved in polyketide synthesis, including CL3211.Contig2_All, Unigene3258_All, Unigene3306_All, CL2002.Contig3_All and CL2002.Contig2_All, than the sucrose-cultured. Tan et al. [17] found that the decrease in citrinin production caused by high concentration of ethanol might be related to the suppression of polyketide pathway. Thus, we postulate that activation of polyketide might be one of the reasons for higher citrinin production by the glucose-cultured *P. citrinum* as compared with that by the sucrose-cultured *P. citrinum*.

Among genes involved in secondary metabolism, oxidoreductase genes seem more critical. Oxidoreductase belongs to the short-chain dehydrogenase/reductase superfamily [42] and may work together with polyketide synthase (PKS) in the polyketide biosynthesis pathway [17]. He et al. [12] also showed that *oxidoreductase* genes are involved in citrinin biosynthesis and clustered with the *PKS* genes. In this study, expressions of most of the oxidoreductase genes were consistent with change in citrinin production between glucose- and sucrose-cultured *P. citrinum*. Thus, we assume that the up-regulation of oxidoreductase may contribute to the high citrinin production by the glucose-cultured *P. citrinum*. However, whether all these oxidoreductase genes are involved in citrinin biosynthesis still needs confirmation in our further study.

As reported previously, the de novo biosynthesis of natural polyketide products and saturated fatty acids share many similarities, including the utilization of same precursors, as well as similar chemistry and structures [43]. Additionally, the inhibition of fatty acid synthase by ethanol treatment on *Monascus purpureus* contributed to the decrease in citrinin production, and therefore, fatty acids might be used as substrates for polyketide syntheses [17]. Our data indicated that most of the genes involved in fatty acid metabolism, including malonyl CoA-acyl carrier protein transacylase (Unigene1634_All), showed higher expressions in the glucose-cultured sample than in the sucrose-cultured sample. The induction of fatty acid biosynthesis by glucose might result in the accumulation of malonyl-CoA or fatty acid. Therefore, our results indicated that there may be a kind of correlation between fatty acid metabolism and citrinin production, which was consistent with what has been previously reported [15]. However, the direct involvement of the fatty acid metabolism in citrinin production needs to be further investigated.

### 3.3. DEGs Involved in Primary Metabolism

Glucose is the major carbon and energy source for most cells [44]. Proteomic characterization of *S. cerevisiae* in response to high glucose concentrations revealed that most of the proteins involved in glycolysis and pentose phosphate pathways were up-regulated [45]. Other previous studies also indicated that the change in primary metabolism could lead to a change in precursor levels and then affect mycotoxin biosynthesis [17,25]. Citrinin has been identified as a polyketide, which begins with the condensation of acetyl-CoA as a precursor. Glycolysis is the metabolic pathway that converts glucose into pyruvate, which can be further metabolized into acetyl-CoA. After entering the TCA cycle, acetyl-CoA is firstly converted into citrate by citrate synthase. In this study, enolase, glucose-6-phosphate 1-epimerase, Glyceraldehyde-3-phosphate dehydrogenase and citrate synthase were up-regulated in the glucose-cultured *P. citrinum* (Figure 2C), suggesting that glucose may promote the TCA cycle. Considering the upregulation of malate dehydrogenase in the glucose-cultured *P. citrinum*, we postulated that an increased amount of carbon might pass through acetyl-CoA during growth of *P. citrinum*. Taking into account the down-regulation of genes involved in ATP generation, it is possible that the *P. citrinum* may have less requirement for energy resources under the glucose condition. Moreover, *P. citrinum* may use acetyl-CoA as precursors for synthesis of polyketides rather than using acetyl-CoA for energy synthesis through the TCA cycle under the glucose condition. This may contribute to the increased citrinin production. Additionally, the upregulation of *alcohol dehydrogenase 2* (CL3211.Contig2_All and Unigene4605_All) and *aldehyde dehydrogenase* (Unigene5443_All) was also found in this study, which could accelerate the formation of acetate. Acetate can be changed into polyketide products through repeated condensation. These results were consistent with previous findings that the decline in acetate production might result in the inhibition of citrinin biosynthesis in *M. purpureus* NTU 568 [17].

A previous report suggested that amino acids presented in the culture medium could control citrinin production [16]. In this study, the genes (Unigene8709_All, Unigene7555_All) involved in glutamate biosynthesis were up-regulated in the glucose-cultured *P. citrinum* as compared with those in the sucrose-cultured *P. citrinum*. However, *proline dehydrogenase* (Unigene125_All), *Pyrroline-5-carboxylate reductase* (Unigene9320_All) and *binuclear zinc transcription factor* (CL1658.Contig9_All) were down-regulated in the glucose-cultured *P. citrinum*, which might induce the accumulation of proline in the medium. Previous research suggested that using glutamate, alanine and proline as the sole nitrogen source resulted in a high level of citrinin [16]. In addition, most of the genes (CL3524.Contig2_All: Branched-chain-amino-acid aminotransferase, Unigene5443_All: Aldehyde dehydrogenase and Unigene4821_All: 6-phosphogluconate dehydrogenase) involved in valine and leucine degradation were up-regulated in the glucose-cultured *P. citrinum*. Tan et al. demonstrated that degradation of branched-chain amino acids facilitated accumulation of methyl-malonyl-CoA, which acted as a precursor in the synthesis of polyketides [17]. Moreover, Hajjaj et al. also showed that valine and leucine had a negative effect on citrinin production [16]. Barad et al. [46] indicated that amino acid metabolism was related to ammonia accumulation which could activate the expression of *pacC*, then contribute to the accumulation of patulin. Nevertheless, we did not identify the differentially expressed *pacC* gene according to this study. In order to confirm the role of *pacC* in the regulation of citrinin biosynthesis, qRT-PCR was conducted to analyze the expression level of the *pacC* gene (annotated in our transcriptome data). We found that *pacC* was slightly up-regulated by glucose (Supplementary Materials Figure S9), which suggested that amino acid metabolism in this study might influence citrinin biosynthesis via regulating the accumulation of the precursor without inducing *pacC* expression. This is different from what Barad et al. reported on patulin biosynthesis [46]. Therefore, the degradation of valine and leucine in the medium with the glucose as the sole carbon source might contribute to high citrinin production derived from the accumulation of the precursor. Regarding the role of *pacC* in regulating secondary metabolism in *P. citrinum*, it may require further investigation. In summary, our data showed that citrinin biosynthesis by *P. citrinum* was closely related to amino acid metabolism.

### 3.4. Signal Transduction and Transcription Factors (TFs)

The signal transducing mechanism plays an important role in understanding various roles of ecophysiological factors influencing mycotoxin biosynthesis [47]. MAPK (mitogen-activated protein kinase) cascades are evolutionarily conserved signaling mechanisms in eukaryotic cells in response to diverse stimuli [48]. HOG (high osmolarity glycerol)-type MAP kinase is also involved in different stress responses of *Fusarium proliferatum* [49]. Jiménez-Martín et al. reported the involvement of the HOG pathway in response of *S. cerevisiae* to high glucose concentrations [44]. Higher levels of Hog1p phosphorylation were found to correlate with higher glucose concentrations [50]. In our study, MAPK signaling pathway genes show different expression patterns between glucose- and sucrose-cultured *P. citrinum*. Although expressions of some genes were induced by glucose, the genes located upstream of MAPK signaling such as sensor histidine kinase SLN1 (Unigene3849_All and Unigene5620_All), and MAP kinase kinase (CL1389.Contig1_All) were down-regulated in the glucose-cultured *P. citrinum*. We assume that the MAPK signaling pathway was inhibited when glucose was used as the sole carbon source. The roles of MAPK signaling in different mycotoxin biosynthesis were different. For example, the disruption of FgOs2 (a HOG-type MAPK orthologue), FgOs4 (encoding a MAPK kinase) or FgOs5 (encoding a MAPK kinases kinase) prevented trichothecene production by *F. graminearum* but greatly increased aurofusarin production [51]. Kohut et al. found that the absence of an intact MAPK pathway could increase the sensitivity of *F. proliferatum* to N-starvation, leading to the upregulation of fumonisin biosynthesis genes and increase in fumonisin production [47]. A previous study also showed that the production of ochratoxin, not citrinin, by *P. verrucosum* was regulated by the HOG MAP kinase pathway and high production of ochratoxin A by *P. nordicum* was consistent with a phosphorylation status of HOG [52]. In addition, HOG phosphorylation can also regulate the shift from citrinin towards biosynthesis of ochratoxin A by *P. verrucosum* under high NaCl concentrations [50]. Taken together, we postulated that the induction of citrinin production by glucose might be related to the reduction in ochratoxin biosynthesis, which may be caused by the inhibition of MAPK signaling under oxidative stress. Schmidt-Heydt et al. also suggested that oxidative stress induced citrinin biosynthesis by *P. verrucosum* at the expense of ochratoxin [36]. However, further research is needed to confirm whether it is the same case in *P. citrinum* as that in *P. verrucosum*.

In the citrinin biosynthesis pathway, the transcriptional activator (ctnA) is one of the important components in the citrinin cluster [53]. According to our qRT-PCR analysis (Figure 3B), the expression level of *ctnA* was higher in the glucose-cultured *P. citrinum* than in the sucrose-cultured *P. citrinum*. We also found that other Zn(II)2Cys6 transcription factors were up-regulated in the glucose-cultured *P. citrinum*. The Zn(II)2Cys6 binuclear cluster DNA-binding domain has been exclusively identified in fungi and generally characterized as transcriptional regulators [54]. Zn(II)2Cys6 transcription factors were also involved in the biosynthesis of secondary metabolites in some fungi including melanin in *Colletotrichum lagenarium* and *Magnaporthe grisea* [55] and fumonisin B_1_ in *F. verticillioides* [56]. Moreover, we found that GATA transcription factor (*areA*), a nitrogen regulatory protein, was down-regulated in the glucose-cultured *P. citrinum*. The *areA* gene encodes a GATA zinc finger transcription factor, activating the expression of many nitrogen metabolite repression related genes [57]. LaeA, another fungal global regulator, has been proven to play an essential role in the regulation of secondary metabolism [26], including the polyketide biosynthesis in *P. citrinum* [58]. Although the present study did not identify the differentially expressed *LaeA* gene in the DEGs, our qRT-PCR results found that all the *LaeA* genes annotated in our transcriptome data were up-regulated in the glucose-cultured *P. citrinum* (Supplementary Materials Figure S9). These results are consistent with a previous report that LaeA was important in regulating patulin production in *P. expansum* [26]. Therefore, besides the specific transcription regulation of the citrinin biosynthetic pathway, basic transcriptional regulation might also be involved in the citrinin biosynthesis.

## 4. Conclusions

**Figure 4:**
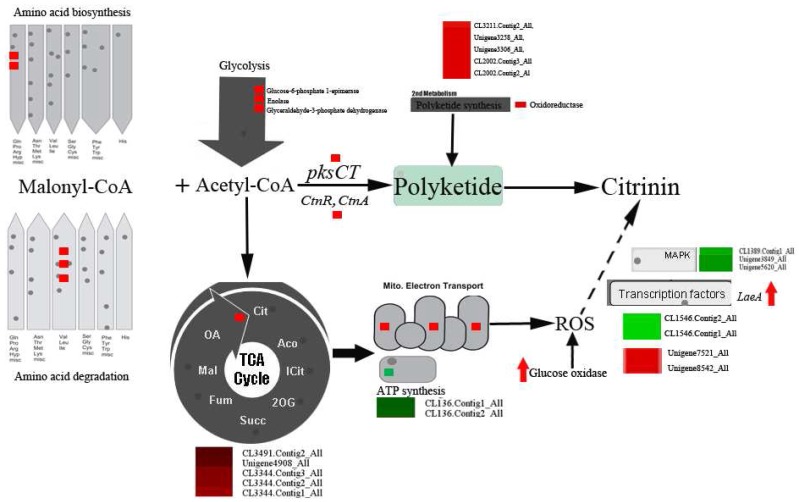
The principal pathways and expression profiles of DEGs involved in citrinin biosynthesis by *P. citrinum*. Dotted arrow indicates that ROS (reactive oxygen species) might regulate citrinin biosynthesis indirectly. Red color indicates up-regulated genes while green indicates down-regulated genes in glucose-cultured *P. citrinum* compared with those in sucrose-cultured *P. citrinum*.

In this study, the molecular basis of citrinin biosynthesis by *P. citrinum* cultured with different carbon sources was investigated via transcriptomic analysis of DEGs. The results from this study are summarized in Figure 4. As compared with the sucrose-cultured *P. citrinum*, the glucose-cultured *P. citrinum* changed its primary metabolic pathways, with more carbon passing through acetyl-CoA and malonyl-CoA, resulting in increased levels of precursors for polyketide synthesis. Additionally, the PKS involved in secondary metabolism and citrinin biosynthesis were up-regulated in the glucose-cultured *P. citrinum*, and led to significantly increased citrinin production. A fungal culture medium containing glucose as the sole carbon source inhibited energy production and activated the ETC process, resulting in ROS formation by *P. citrinum*. Further, up-regulation of *GOX* under the glucose culture also contributed to higher H_2_O_2_ content in *P. citrinum.* In response to oxidative stress, *P. citrinum* might produce a higher level of citrinin by altering expression levels of signaling pathway genes, antioxidant enzymes, etc., via basic transcriptional regulation. Finally, more protein degradation might occur in the glucose-cultured *P. citrinum* than in the sucrose-cultured *P. citrinum* in response to redox stress. However, the exact relationship between ROS and citrinin biosynthesis is worthy of further investigation. Overall, this study presents useful knowledge for understanding pathways and regulations of citrinin biosynthesis by *P. citrinum* at the molecular level.

## 5. Materials and Methods

### 5.1. Fungal Strain and Growth Condition

*P. citrinum* (AS3.458, purchased from Guangdong Microbiology Culture Center, Guangzhou, China) originally stored in 50% glycerol at −80 °C, was cultured at 28 °C on potato dextrose agar (PDA) plates (Oxoid, Basingstoke, Hampshire, UK) for 7 days. Then, six small plates (5 mm) were cut and transferred to the Czapek’s broth medium (3.0 g/L NaNO_3_, 1.0 g/L K_2_HPO_4_, 0.5 g/L MgSO_4_·7H_2_O, 0.5 g/L KCl, 0.01 g/L FeSO_4_) with sucrose (30 g/L) or glucose (30 g/L) as carbon sources, respectively. Both sucrose and glucose were purchased from Gen-view Scientific Inc. (Calimesa, CA, USA). The conical flasks, each containing 100 mL of the above culture broth, were incubated at 28 °C with shaking at 200 rpm for 7 days. After that, the cultured broth was filtered off at a vacuum pump and the fungal biomass was determined. The mycelia of each biological replicate was washed thoroughly with cold sterile distilled water, quickly frozen in liquid nitrogen, and then stored at −80 °C still the experimental use. The filtrate was collected to determine citrinin production. Three biological replicates were conducted for citrinin analysis and two biological replicates were performed for RNA-Seq.

### 5.2. Citrinin Analysis

The citrinin was extracted according to the method described by Li et al. [11]. Briefly, the filtrate was extracted by methanol. Then, the solution was filtered through a 0.22-μm Millipore membrane filter before the ultra-performance liquid chromatography (UPLC)-mass spectrometry (MS)/MS analysis. The citrinin concentration was determined by an AB-SCIEX TRIPLE QUADTM 5500 UPLC-MS/MS system (AB SCIEX, Redwood City, CA, USA). The obtained sample (10 μL) was injected into an UPLC column (C18 column, 100 × 2.1 mm, 3 µm particle size, Thermo, Waltham, WI, USA). An optimized gradient of mobile phase (A: acetonitrile and B: 5 mM ammonium acetate) was used. Volume fraction of A was kept at 20% for 10 s, increased linearly to 80% in 10 min and then kept constant for 50 s. Finally, the volume fraction of A was decreased to 20% and kept constant for 10 s. The flow rate was set at 0.6 mL/min. N_2_ was used as a nebulizing gas at a nebulizer temperature of 450 °C. Positive ionization was selected for mass spectrometric detection. A multiple reaction monitoring (MRM) function was employed for quantification of citrinin, with the parent ions at *m*/*z* 251. The MS conditions were optimized for citrinin quantification, including declustering potential (49 V), entrance potential (10 V), collision exit potential (14 V), collision energy (22 eV) and ion spray voltage (5500 V). The detection limit was 0.1 ng·mL^−1^. Commercial citrinin (Sigma, St. Louis, MO, USA) was used as standard.

### 5.3. RNA Extraction, Library Construction and Sequencing

Total RNA was isolated from the mycelia of 7-day-old *P. citrinum* grown in Czapek’s broth medium with different carbon sources. Two replicates were performed. The total RNA was extracted using the Hipure Fungal RNA Mini Kit (Magen, Shanghai, China) in accordance with the manufacturer’s instructions and then stored at −80 °C. RNA purity was checked by a NanoPhotometer^®^ spectrophotometer (Implen, Westlake Village, CA, USA). RNA integrity was assessed using the RNA 6000 Nano Assay Kit with the Bioanalyzer 2100 system (Agilent Technologies, PaloAlto, CA, USA). The purified total RNA samples were sent to Hengchuang Co. Ltd. (Shenzhen, China), where the samples were further processed and a cDNA library for each sample was constructed. Briefly, the mRNA was enriched using the oligo (dT) magnetic beads, which was then fragmented into short fragments and mixed with the fragmentation buffer (The NEB Next Multiplex Oligos for Illumine, NEB, Ipswich, MA, UK). The first and second strands of cDNA were synthesized by using random hexamer-primer and DNA polymerase I, which were provided by Illumina, Inc. (San Diego, CA, USA). After the 3′-end single nucleotide A (adenine) and adaptor were added to each cDNA fragment, their quality and quantity were determined using Agilent 2100 Bioanaylzer (Palo Alto, Cal. USA). Finally, each library was sequenced with Illumina HiSeq™ 2000 (San Diego, CA, USA) using the paired-end technology.

### 5.4. *De Novo* Assembly and Annotation of the P. citrinum Unigenes

Clean reads were obtained after removing the raw reads containing the adapter, reads containing more than 5% unknown nucleotides, and low quality reads containing more than 20% low quality (Q value ≤ 10) bases. Then, the clean reads were de novo assembled by the Trinity program into contigs [59], which were further processed with TIGR Gene Indices clustering tools (TGICL, v2.1) to effectively remove redundancy [60]. The unigenes generated were annotated using BLASTx program [61] with an E-value threshold of 1 × 10^−5^ to NCBI Nr database [62], the Swiss-Prot protein database [63] the KEGG database [64] and the COG database [65]. GO annotations were conducted using Blast2Go program [66] and KEGG pathway annotation was performed according to the method described by Kanehisa et al. [67].

### 5.5. DEGs between Different Samples and Function Enrichment

Clean reads were mapped to reference sequence by the SOAP (Release 2.21), a tool designed for short sequences alignment. Reads uniquely mapped to unigenes were used to calculate the expression level, expressed as Fragments Per kb per Million fragments (FPKM). After the expression level of each gene was calculated, differential expression analysis was conducted using the method described by Audic et al. [68]. The false discovery rate was used to determine the threshold of the *p*-value in multiple tests. For the analysis, the thresholds FDR ≤ 0.001 and |log2Ratio| ≥ 1 were used to determine the significance of differences in gene expression. The differentially regulated DEGs between two samples were separately subjected to GO [69] with corrected *p*-value ≤ 0.05 and KEGG pathway enrichment with Q value ≤ 0.05.

### 5.6. Evaluation of Genes Expression by qRT-PCR

On the basis of known sequences, primers of 49 genes for quantitative real-time PCR (qPCR) were designed using Primer Premier 6.0 software (Premier, Canada) and synthesized by Sangon Biotech Co., Ltd. (Shanghai, China). Sequence information of all the primers is shown in Supplementary Materials Table S3. The expression level of *Histone H3* (CL2661.Contig1_All) was used as the standard to normalize the content of cDNA and the 2^−ΔΔCt^ method. SYBR (Synergy Brand) Premix Ex Taq™ mix (TaKaRa, Dalian, China) was used as the reaction mixture with further addition of 10.0 µL of SYBR Premix Ex Taq™, 0.4 µL of PCR forward primer (10 µM), 0.4 µL of PCR reverse primer (10 µM), 0.4 µL of ROX reference dye II and 2 µL (20 ng) of cDNA (a final volume of 20 µL). The qPCR was performed with a 7500 Fast Real-Time PCR System (Applied Biosystems, Foster City, CA, USA). The 7500 Fast System Software 2.0.1 was used to analyze data in fluorescent intensity. Amplification conditions were 40 cycles at 95 °C for 30 s, 90 °C for 5 s and 72 °C for 34 s. The comparative Ct (ΔΔCT) method was performed to determine the relative change in the genes expression.

### 5.7. Measurement of H_2_O_2_ Contents

An H_2_O_2_ assay kit (Jiancheng Bioengineering Institute, Nanjing, China) was used to analyze the H_2_O_2_ content. Specifically, 0.1 g of the sample was ground into powder in the presence of liquid nitrogen, and then 0.9 mL of extraction buffer was added to it. After being mixed with a vortex for 1 min, the sample was centrifuged for 10 min at 10,000 rpm. Then, the obtained supernatants were used for detecting H_2_O_2_ content according to the manufacturer’s instructions.

### 5.8. Statistical Analyses

Three biological replicates were used for each sample for citrinin content, H_2_O_2_ content and qPCR analysis. Data for each sample were statistically analyzed using Student’s *t*-test (*p* < 0.05).

## Figures and Tables

**Table 1:** GO (Gene ontology) enrichment analysis of DEGs (Differentially expressed genes).

GO Term	Ontology *	Description	Number of Genes	Corrected-*p*-Value
GO: 0098656	BP	anion transmembrane transport	29	0.00010
GO: 0003333	BP	amino acid transmembrane transport	25	0.00024
GO: 0006734	BP	NADH metabolic process	6	0.0004
GO: 0006116	BP	NADH oxidation	5	0.00179
GO: 0006865	BP	amino acid transport	25	0.00198
GO: 0071705	BP	nitrogen compound transport	40	0.00532
GO: 0015862	BP	uridine transport	4	0.00619
GO: 0015864	BP	pyrimidine nucleoside transport	4	0.00619
GO: 0015849	BP	organic acid transport	27	0.00983
GO: 0046942	BP	carboxylic acid transport	27	0.00983
GO: 0055114	BP	oxidation-reduction process	115	0.02007
GO: 0015711	BP	organic anion transport	29	0.02227
GO: 0006072	BP	glycerol-3-phosphate metabolic process	6	0.03336

* Biological Process (BP).

**Table 2:** KEGG (Kyoto Encyclopedia of Genes and Genomes) pathway enrichment analysis of DEGs.

Pathway ID	KEGG Pathway	Number of Genes	Q-Value
ko01110	Biosynthesis of secondary metabolites	123	0.003588954
ko01100	Metabolic pathways	255	0.003588954
ko00630	Glyoxylate and dicarboxylate metabolism	18	0.008545071
ko00260	Glycine, serine and threonine metabolism	25	0.039994923
ko00100	Steroid biosynthesis	17	0.039994923
ko00061	Fatty acid biosynthesis	11	0.039994923

## Supplementary Materials

The following are available online at www.mdpi.com/2072-6651/9/2/69/s1. Table S1. Summary of RNA-Seq data. S-samples were grown with sucrose and the G-Samples were grown with glucose as sole carbon source; Table S2. Results of de novo assembly; Table S3. The primer sequences of 49 unigenes for qRT-PCR in this study; Figure S1. Number of the annotated genes against different databases; Figure S2. (A) E-value distribution of unigenes annotated in the nr database. (B) Species distribution of the first BLAST hits for each sequence with a cut-off E-value of 1.0E-5.; Figure S3. Gene Ontology (GO) classification of all unigenes. All unigenes were classified with respect to biological processes, molecular functions and cellular components; Figure S4. COG function classification of all unigenes; Figure S5. Spearman correlation analysis of the two replicates in each group. The vertical and horizontal axes represent the expression levels of all unigenes (log10 (FPKM)); Figure S6. Cluster analysis of DEGs according to GO analysis. A total of 1085 DEGs were clustered using Blast2GO, and the analysis was performed with respect to biological processes, molecular functions and cellular components; Figure S7. Cluster analysis of DEGs according to KEGG pathways. All those genes were classified according to the KEGG pathway database. Clusters of interest were selected for subsequent analysis; Figure S8. Expression profiles of DEGs involved in transcription factors; Excel S1.
