# Supplementary material for: Comparative Transcriptome Analysis of Penicillium citrinum Cultured with Different Carbon Sources Identifies Genes Involved in Citrinin Biosynthesis
Source: Toxins (Basel). 2017 Feb 21;9(2):69. doi: 10.3390/toxins9020069 (PMC5331448; doi:10.3390/toxins9020069)
Supplement: Supplementary file 1 [file toxins-09-00069-s001.zip › toxins-166702-suppl/toxins-166702 supply.pdf]

Supplementary Materials: Comparative transcriptome analysis of *Penicillium citrinum* cultured with different carbon sources identifies genes involved in citrinin biosynthesis

Taotao Li, Guoxiang Jiang, Hongxia Qu, Yong Wang, Yehui Xiong, Qijie Jian, Yu Wu, Xuewu Duan, Xiangrong Zhu, Wenzhong Hu, JiashengWang, Liang Gong and Yueming Jiang

Table S1. Summary of RNA-Seq data. S-samples were grown with sucrose and the G-Samples were grown with glucose as sole carbon source.

Sample name	Total reads	Total nucleotide (nt)	Q>=20	Adapter	Low quality
S1	41024262	6153639300	95.76%	10.27%	0.02%
S2	40904432	6135664800	95.24%	7.22%	0.02%
G1	40267398	6040109700	95.95%	8.66%	0.02%
G2	41064036	6159605400	95.68%	10.18%	0.02%

Table S2. Results of *de novo* assembly.

Unigene	N50	Average length	Total assembled bases
25491	3889	2048	52211935

Table S3. The primer sequences of 49 unigenes for qRT-PCR in this study.

Gene ID	Sequence of primer (5′ to 3′)
Unigene4605_All	For: ACTCATTCTCGCACGCTGGATGT<br>Rev: TCTCTTCTCAGCCGCCAACTTCAA
Unigene5443_All	For: CTTGGTGGCGATGCGAATGATGG<br>Rev: CTTCTGAATAGCCTCTTCCTCCGTCTT
Unigene4470_All	For: AATGCTGGCGATGCTGGTATGGA<br>Rev: GTGGCTGCTGGTGAACTAGACGAT
Unigene1201_All	For: ATCTGATCGGTCGCATCCGTCTG<br>Rev: ACCTATAAGCCTCCTCGTCCTTCGT
Unigene3306_All	For: GGAACTTGGAACCTTCATCACGCATT<br>Rev: ACGGACGCAGGAAGCCCTTTATC
CL2002.Contig3_All	For: CGCTTCCGCTATGATCCGACCAT<br>CAACAAGACCTATTTCAACGCCGAGAT
CL2002.Contig2_All	For: CGCTTCCGCTATGATCCGACCAT<br>Rev: CAACAAGACCTATTTCAACGCCGAGAT
CL3211.Contig2_All	For: ACCTGACGATGTACTTATCCGACTCA<br>Rev: GTCTCCTTATCATTCCAGCACATAGCA
Unigene3258_All	For: GAGATGGTGTCCGTGATTGCTGAG<br>Rev: GTATTGTTCGTAGCCATAGTCACTCCTC
CL1938.Contig2_All	For: GTCAAATCCAGCCTGAGGAAAGTGT<br>TGGTCGCCATTATGAGTCAACAACAAT
CL1093.Contig2_All	For: TCCAAGTATCAAGTGGTCTAGGCTCAT<br>Rev: CTTCGGAATCTCGCTCTCCTTCAAG
CL2531.Contig1_All	For: AACAATCGCTTACGCCTGCTTACA<br>Rev: GAGCCTTGAGCACTATATCAACCACTT
Unigene950_All	For: GACTCGGTGGCTGGAGATGGAT<br>Rev: GTGGTGAGACAGAAGAAGATTAGAGCAA
Unigene401_All	For: ATCTCCAACTTGCCATTGCCATCA<br>Rev: CTCTCCGTCCGAGCATCCAGAA
Unigene6956_All	For: CCTGATGACGAGTTCAAGCCTGAC<br>Rev: GATCACCATTGCCGCTGTAGACAT
Unigene1095_All	For: AGACAGCCAGAAGAGTGAAGGAGAG<br>CGACATCAGTTGACTTGGACCGTATT
CL1869.Contig6_All	For: TTCAACGGCAGTCCTATCATCATCATT<br>Rev: GCCTCCATAATTAACACTGTCCTTCCA
Unigene3849_All	For: GGACGAACCACACCGCATTCTATT<br>Rev: TTCCACGCCTCAAGAGCTTCCT
Unigene5620_All	For: CCGTGCGAATTACCATAGCCGATT<br>Rev: CGAGACTCAATCCTACTCCAAGACCT
Unigene8542_All	For: GGAACTTCCGCACTCTGATGTATCG<br>Rev: CTGCTGGACACTGTGGATCATTACG
CL2339.Contig4_All	For: AGACTGCTGAGGAGGCTCTTGAC<br>Rev: GTAGAACTCGCTGGAGGCAACATC

Unigene548_All	For: CCGCCGTGCCGATTATCATCTC<br>Rev: CCAGCCTCAACACAGAGCATCTTC
Unigene6764_All	For: ATTCTTCGGCTTCATTGAGTCCTTCC<br>Rev: CCAGCAGATAAGAGAGCGTAGAGAGTA
Unigene2457_All	For: GACCGCTGATCTGATTGATTCACCTT<br>Rev: TCGTTAGGCTGATGTGTACCATTGC
CL2164.Contig11_All	For: CGACACCGACCGCAAGTTCTAC<br>Rev: ACCATCACCGCCCAGAGTAGTC
Unigene11940_All	For: GGCTGAGTTATATGGCGAAGATGGTATT<br>Rev: TTAGGCAAGGCATCTGGTAGAGGAA
Unigene5219_All	For: CTCGTTGGTAGTGTCAAGGATGTTAAGT<br>Rev: GCTGCTTCAGTTCGCTGCTGTA
CL1796.Contig2_All	For: TCAACACGCTATCATTCCTCGCTAAG<br>Rev: GCCTTCCCATTGCCATTGTCATTTC
CL1031.Contig3_All	For: TCGGTTGGACTCTACAGCAAGGAT<br>Rev: CGGTGGTGAAGTATCTGAACGGATG
Unigene8156_All	For: GAACAGGAACTCCCGTCGGTGA<br>Rev: CTGAAGGGTGCTTGCCAGTCTTT
CL1031.Contig5_All	For: GATGCGGCTTTCACCTCACTACTT<br>Rev: TTGTCTGACCAGCTCCATTGATGC
CL348.Contig2_All	For: GGAACTTACAACAGCACTCACCAGAAC<br>Rev: CCAATCCACCAACACCAATGATACCAA
Unigene125_All	For: AAGCACACCATCTACAAGCAGTTCAA<br>Rev: TTCACGAGCATAGCCAAGGAGGA
CL1559.Contig1_All	TCGGCACACCTGCGTACTTCAATA<br>CGGTAGCAATGACATCAACATCCACAG
Unigene13097_All	CAGGAGACTCGTGCCAATCAGACA<br>AGATGGGTGGTACACTTGCGTCAG
Unigene2872_All	GGCACTAGGCGGACCACTTGAT<br>GCCAATCTTCATCTTCCAATCCAGCTC
Unigene4460_All	CACCGTTGCTTCTAAACTCACCGAAA<br>TTCCATTGACCAGTGTAGAGCCTCC
Unigene5036_All	CCGTGAGCACTTGTTCCATGATGAC<br>GCAGAAACCTGGGTAGGAGGAATAGAC
Unigene5396_All	TCTTGTTGGCTGCTGGATCTTCTGT<br>GAGGAGGTCAGTTGCGTAGTCAGTT
CL1213.Contig1_All	CGGCTTCTGAAGTCGTTGGGATTG<br>GCTCTGAGGTGAGTATCATCATCGGAAT
CL1213.Contig2_All	CCCAACGATGAGCGAGAGCAAGA<br>CCCATATCCCTGTACCAGTCCCTAAATC
CL1213.Contig6_All	TCAGCATACAATGGCTCGATAAGCAATG<br>TCTCGCTCATCGTTGGGCAAGAA
Unigene2855_All	GAGCCAGCGGTCATCACCAAGA<br>GCAAGTAGCCCAACCCTCGTGTA

CL2103.Contig1_All	GCGAAGTCGAGGAGAATGTAGAGTATGT<br>GCAATAATGTCTTCACCGCTGTCACTAT
CL2103.Contig5_All	TTCTGGACGAGTTGATGGTACTGTTGT<br>CCTGAGAGATTGAATTTGGCGAGTGTG
CL2103.Contig6_All	ACCTAGCTTCTGGACGAGTGAATACG<br>TGAGAGATTGAATTTGGCGAGTGTGAC
CL1659.Contig3_All	CTGAGCAAGACAGCAAGACTGAAGATG<br>CTATGCGACGAAGGTTCTCCAGAAGA
Unigene7245_All	ACGTGCAACTCAAGGACTCTCAGC<br>ATTTGGCCGTGAAGGACGACCA
CL1659.Contig1_All	GGCGTTGATAAGCGGTCGTATGC<br>CATCCTTAGCGGTAGGCGAGTCAG
CL2661.Contig1_All	For: TCCGTGAGATCCGTCGCTACCA<br>Rev: ACTCCTGAAGAGCACCGATGGC

Gene ID	Sequence of primer (5′ to 3′)
Unigene4605_All	For: ACTCATTCTCGCACGCTGGATGT<br>Rev: TCTCTTCTCAGCCGCCAACTTCAA
Unigene5443_All	For: CTTGGTGGCGATGCGAATGATGG<br>Rev: CTTCTGAATAGCCTCTTCCTCCGTCTT
Unigene4470_All	For: AATGCTGGCGATGCTGGTATGGA<br>Rev: GTGGCTGCTGGTGAACTAGACGAT
Unigene1201_All	For: ATCTGATCGGTCGCATCCGTCTG<br>Rev: ACCTATAAGCCTCCTCGTCCTTCGT
Unigene3306_All	For: GGAACTTGGAACCTTCATCACGCATT<br>Rev: ACGGACGCAGGAAGCCCTTTATC
CL2002.Contig3_All	For: CGCTTCCGCTATGATCCGACCAT<br>CAACAAGACCTATTTCAACGCCGAGAT
CL2002.Contig2_All	For: CGCTTCCGCTATGATCCGACCAT<br>Rev: CAACAAGACCTATTTCAACGCCGAGAT
CL3211.Contig2_All	For: ACCTGACGATGTACTTATCCGACTCA<br>Rev: GTCTCCTTATCATTCCAGCACATAGCA
Unigene3258_All	For: GAGATGGTGTCCGTGATTGCTGAG<br>Rev: GTATTGTTCGTAGCCATAGTCACTCCTC
CL1938.Contig2_All	For: GTCAAATCCAGCCTGAGGAAAGTGT<br>TGGTCGCCATTATGAGTCAACAACAAT
CL1093.Contig2_All	For: TCCAAGTATCAAGTGGTCTAGGCTCAT<br>Rev: CTTCGGAATCTCGCTCTCCTTCAAG
CL2531.Contig1_All	For: AACAATCGCTTACGCCTGCTTACA<br>Rev: GAGCCTTGAGCACTATATCAACCACTT
Unigene950_All	For: GACTCGGTGGCTGGAGATGGAT<br>Rev: GTGGTGAGACAGAAGAAGATTAGAGCAA
Unigene401_All	For: ATCTCCAACTTGCCATTGCCATCA<br>Rev: CTCTCCGTCCGAGCATCCAGAA
Unigene6956_All	For: CCTGATGACGAGTTCAAGCCTGAC<br>Rev: GATCACCATTGCCGCTGTAGACAT

Unigene1095_All	For: AGACAGCCAGAAGAGTGAAGGAGAG<br>CGACATCAGTTGACTTGGACCGTATT
CL1869.Contig6_All	For: TTCAACGGCAGTCCTATCATCATCATT<br>Rev: GCCTCCATAATTAACACTGTCCTTCCA
Unigene3849_All	For: GGACGAACCACACCGCATTCTATT<br>Rev: TTCCACGCCTCAAGAGCTTCCT
Unigene5620_All	For: CCGTGCGAATTACCATAGCCGATT<br>Rev: CGAGACTCAATCCTACTCCAAGACCT
Unigene8542_All	For: GGAACTTCCGCACTCTGATGTATCG<br>Rev: CTGCTGGACACTGTGGATCATTACG
CL2339.Contig4_All	For: AGACTGCTGAGGAGGCTCTTGAC<br>Rev: GTAGAACTCGCTGGAGGCAACATC
Unigene548_All	For: CCGCCGTGCCGATTATCATCTC<br>Rev: CCAGCCTCAACACAGAGCATCTTC
Unigene6764_All	For: ATTCTTCGGCTTCATTGAGTCCTTCC<br>Rev: CCAGCAGATAAGAGAGCGTAGAGAGTA
Unigene2457_All	For: GACCGCTGATCTGATTGATTCACCTT<br>Rev: TCGTTAGGCTGATGTGTACCATTGC
CL2164.Contig11_All	For: CGACACCGACCGCAAGTTCTAC<br>Rev: ACCATCACCGCCCAGAGTAGTC
Unigene11940_All	For: GGCTGAGTTATATGGCGAAGATGGTATT<br>Rev: TTAGGCAAGGCATCTGGTAGAGGAA
Unigene5219_All	For: CTCGTTGGTAGTGTCAAGGATGTTAAGT<br>Rev: GCTGCTTCAGTTCGCTGCTGTA
CL1796.Contig2_All	For: TCAACACGCTATCATTCCTCGCTAAG<br>Rev: GCCTTCCCATTGCCATTGTCATTTC
CL1031.Contig3_All	For: TCGGTTGGACTCTACAGCAAGGAT<br>Rev: CGGTGGTGAAGTATCTGAACGGATG
Unigene8156_All	For: GAACAGGAACTCCCGTCGGTGA<br>Rev: CTGAAGGGTGCTTGCCAGTCTTT
CL1031.Contig5_All	For: GATGCGGCTTTCACCTCACTACTT<br>Rev: TTGTCTGACCAGCTCCATTGATGC
CL348.Contig2_All	For: GGAACTTACAACAGCACTCACCAGAAC<br>Rev: CCAATCCACCAACACCAATGATACCAA
Unigene125_All	For: AAGCACACCATCTACAAGCAGTTCAA<br>Rev: TTCACGAGCATAGCCAAGGAGGA
CL1559.Contig1_All	TCGGCACACCTGCGTACTTCAATA<br>CGGTAGCAATGACATCAACATCCACAG
Unigene13097_All	CAGGAGACTCGTGCCAATCAGACA<br>AGATGGGTGGTACACTTGCGTCAG
Unigene2872_All	GGCACTAGGCGGACCACTTGAT<br>GCCAATCTTCATCTTCCAATCCAGCTC
Unigene4460_All	CACCGTTGCTTCTAAACTCACCGAAA<br>TTCCATTGACCAGTGTAGAGCCTCC

Unigene5036_All	CCGTGAGCACTTGTTCCATGATGAC<br>GCAGAAACCTGGGTAGGAGGAATAGAC
Unigene5396_All	TCTTGTTGGCTGCTGGATCTTCTGT<br>GAGGAGGTCAGTTGCGTAGTCAGTT
CL1213.Contig1_All	CGGCTTCTGAAGTCGTTGGGATTG<br>GCTCTGAGGTGAGTATCATCATCGGAAT
CL1213.Contig2_All	CCCAACGATGAGCGAGAGCAAGA<br>CCCATATCCCTGTACCAGTCCCTAAATC
CL1213.Contig6_All	TCAGCATACAATGGCTCGATAAGCAATG<br>TCTCGCTCATCGTTGGGCAAGAA
Unigene2855_All	GAGCCAGCGGTCATCACCAAGA<br>GCAAGTAGCCCAACCCTCGTGTA
CL2103.Contig1_All	GCGAAGTCGAGGAGAATGTAGAGTATGT<br>GCAATAATGTCTTCACCGCTGTCACTAT
CL2103.Contig5_All	TTCTGGACGAGTTGATGGTACTGTTGT<br>CCTGAGAGATTGAATTTGGCGAGTGTG
CL2103.Contig6_All	ACCTAGCTTCTGGACGAGTGAATACG<br>TGAGAGATTGAATTTGGCGAGTGTGAC
CL1659.Contig3_All	CTGAGCAAGACAGCAAGACTGAAGATG<br>CTATGCGACGAAGGTTCTCCAGAAGA
Unigene7245_All	ACGTGCAACTCAAGGACTCTCAGC<br>ATTTGGCCGTGAAGGACGACCA
CL1659.Contig1_All	GGCGTTGATAAGCGGTCGTATGC<br>CATCCTTAGCGGTAGGCGAGTCAG
CL2661.Contig1_All	For: TCCGTGAGATCCGTCGCTACCA<br>Rev: ACTCCTGAAGAGCACCGATGGC

Gene ID	Sequence of primer (5′ to 3′)
Unigene4605_All	For: ACTCATTCTCGCACGCTGGATGT<br>Rev: TCTCTTCTCAGCCGCCAACTTCAA
Unigene5443_All	For: CTTGGTGGCGATGCGAATGATGG<br>Rev: CTTCTGAATAGCCTCTTCCTCCGTCTT
Unigene4470_All	For: AATGCTGGCGATGCTGGTATGGA<br>Rev: GTGGCTGCTGGTGAACTAGACGAT
Unigene1201_All	For: ATCTGATCGGTCGCATCCGTCTG<br>Rev: ACCTATAAGCCTCCTCGTCCTTCGT
Unigene3306_All	For: GGAACTTGGAACCTTCATCACGCATT<br>Rev: ACGGACGCAGGAAGCCCTTTATC
CL2002.Contig3_All	For: CGCTTCCGCTATGATCCGACCAT<br>CAACAAGACCTATTTCAACGCCGAGAT
CL2002.Contig2_All	For: CGCTTCCGCTATGATCCGACCAT<br>Rev: CAACAAGACCTATTTCAACGCCGAGAT
CL3211.Contig2_All	For: ACCTGACGATGTACTTATCCGACTCA<br>Rev: GTCTCCTTATCATTCCAGCACATAGCA
Unigene3258_All	For: GAGATGGTGTCCGTGATTGCTGAG<br>Rev: GTATTGTTCGTAGCCATAGTCACTCCTC

CL1938.Contig2_All	For: GTCAAATCCAGCCTGAGGAAAGTGT<br>TGGTCGCCATTATGAGTCAACAACAAT
CL1093.Contig2_All	For: TCCAAGTATCAAGTGGTCTAGGCTCAT<br>Rev: CTTCGGAATCTCGCTCTCCTTCAAG
CL2531.Contig1_All	For: AACAATCGCTTACGCCTGCTTACA<br>Rev: GAGCCTTGAGCACTATATCAACCACTT
Unigene950_All	For: GACTCGGTGGCTGGAGATGGAT<br>Rev: GTGGTGAGACAGAAGAAGATTAGAGCAA
Unigene401_All	For: ATCTCCAACTTGCCATTGCCATCA<br>Rev: CTCTCCGTCCGAGCATCCAGAA
Unigene6956_All	For: CCTGATGACGAGTTCAAGCCTGAC<br>Rev: GATCACCATTGCCGCTGTAGACAT
Unigene1095_All	For: AGACAGCCAGAAGAGTGAAGGAGAG<br>CGACATCAGTTGACTTGGACCGTATT
CL1869.Contig6_All	For: TTCAACGGCAGTCCTATCATCATCATT<br>Rev: GCCTCCATAATTAACACTGTCCTTCCA
Unigene3849_All	For: GGACGAACCACACCGCATTCTATT<br>Rev: TTCCACGCCTCAAGAGCTTCCT
Unigene5620_All	For: CCGTGCGAATTACCATAGCCGATT<br>Rev: CGAGACTCAATCCTACTCCAAGACCT
Unigene8542_All	For: GGAACTTCCGCACTCTGATGTATCG<br>Rev: CTGCTGGACACTGTGGATCATTACG
CL2339.Contig4_All	For: AGACTGCTGAGGAGGCTCTTGAC<br>Rev: GTAGAACTCGCTGGAGGCAACATC
Unigene548_All	For: CCGCCGTGCCGATTATCATCTC<br>Rev: CCAGCCTCAACACAGAGCATCTTC
Unigene6764_All	For: ATTCTTCGGCTTCATTGAGTCCTTCC<br>Rev: CCAGCAGATAAGAGAGCGTAGAGAGTA
Unigene2457_All	For: GACCGCTGATCTGATTGATTCACCTT<br>Rev: TCGTTAGGCTGATGTGTACCATTGC
CL2164.Contig11_All	For: CGACACCGACCGCAAGTTCTAC<br>Rev: ACCATCACCGCCCAGAGTAGTC
Unigene11940_All	For: GGCTGAGTTATATGGCGAAGATGGTATT<br>Rev: TTAGGCAAGGCATCTGGTAGAGGAA
Unigene5219_All	For: CTCGTTGGTAGTGTCAAGGATGTTAAGT<br>Rev: GCTGCTTCAGTTCGCTGCTGTA
CL1796.Contig2_All	For: TCAACACGCTATCATTCCTCGCTAAG<br>Rev: GCCTTCCCATTGCCATTGTCATTTC
CL1031.Contig3_All	For: TCGGTTGGACTCTACAGCAAGGAT<br>Rev: CGGTGGTGAAGTATCTGAACGGATG
Unigene8156_All	For: GAACAGGAACTCCCGTCGGTGA<br>Rev: CTGAAGGGTGCTTGCCAGTCTTT
CL1031.Contig5_All	For: GATGCGGCTTTCACCTCACTACTT<br>Rev: TTGTCTGACCAGCTCCATTGATGC
CL348.Contig2_All	For: GGAACTTACAACAGCACTCACCAGAAC<br>Rev: CCAATCCACCAACACCAATGATACCAA

Unigene125_All	For: AAGCACACCATCTACAAGCAGTTCAA<br>Rev: TTCACGAGCATAGCCAAGGAGGA
CL1559.Contig1_All	TCGGCACACCTGCGTACTTCAATA<br>CGGTAGCAATGACATCAACATCCACAG
Unigene13097_All	CAGGAGACTCGTGCCAATCAGACA<br>AGATGGGTGGTACACTTGCGTCAG
Unigene2872_All	GGCACTAGGCGGACCACTTGAT<br>GCCAATCTTCATCTTCCAATCCAGCTC
Unigene4460_All	CACCGTTGCTTCTAAACTCACCGAAA<br>TTCCATTGACCAGTGTAGAGCCTCC
Unigene5036_All	CCGTGAGCACTTGTTCCATGATGAC<br>GCAGAAACCTGGGTAGGAGGAATAGAC
Unigene5396_All	TCTTGTTGGCTGCTGGATCTTCTGT<br>GAGGAGGTCAGTTGCGTAGTCAGTT
CL1213.Contig1_All	CGGCTTCTGAAGTCGTTGGGATTG<br>GCTCTGAGGTGAGTATCATCATCGGAAT
CL1213.Contig2_All	CCCAACGATGAGCGAGAGCAAGA<br>CCCATATCCCTGTACCAGTCCCTAAATC
CL1213.Contig6_All	TCAGCATACAATGGCTCGATAAGCAATG<br>TCTCGCTCATCGTTGGGCAAGAA
Unigene2855_All	GAGCCAGCGGTCATCACCAAGA<br>GCAAGTAGCCCAACCCTCGTGTA
CL2103.Contig1_All	GCGAAGTCGAGGAGAATGTAGAGTATGT<br>GCAATAATGTCTTCACCGCTGTCACTAT
CL2103.Contig5_All	TTCTGGACGAGTTGATGGTACTGTTGT<br>CCTGAGAGATTGAATTTGGCGAGTGTG
CL2103.Contig6_All	ACCTAGCTTCTGGACGAGTGAATACG<br>TGAGAGATTGAATTTGGCGAGTGTGAC
CL1659.Contig3_All	CTGAGCAAGACAGCAAGACTGAAGATG<br>CTATGCGACGAAGGTTCTCCAGAAGA
Unigene7245_All	ACGTGCAACTCAAGGACTCTCAGC<br>ATTTGGCCGTGAAGGACGACCA
CL1659.Contig1_All	GGCGTTGATAAGCGGTCGTATGC<br>CATCCTTAGCGGTAGGCGAGTCAG
CL2661.Contig1_All	For: TCCGTGAGATCCGTCGCTACCA<br>Rev: ACTCCTGAAGAGCACCGATGGC

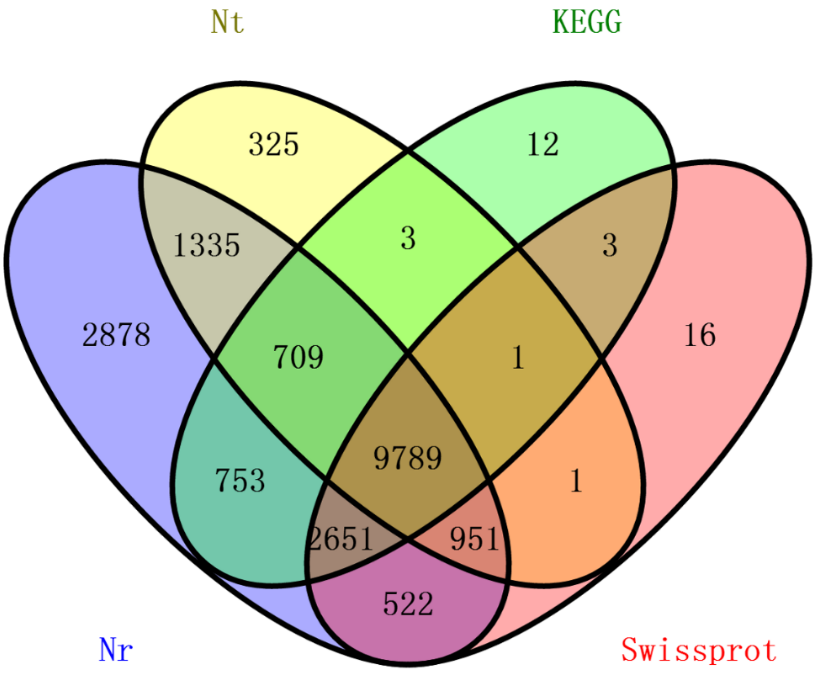

Figure S1. Number of the annotated genes against different databases.

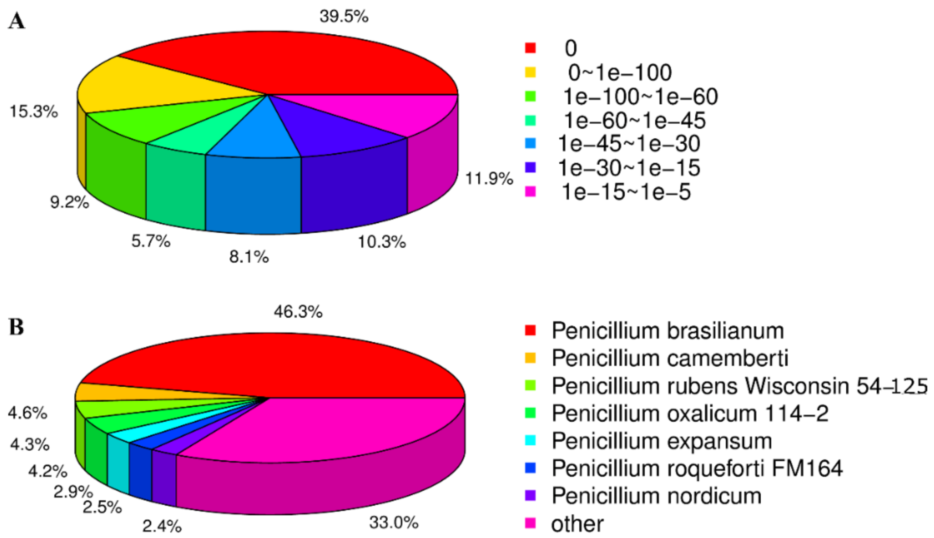

Figure S2. (A) E-value distribution of unigenes annotated in the nr database. (B) Species distribution of the first BLAST hits for each sequence with a cut-off E-value of 1.0E-5.

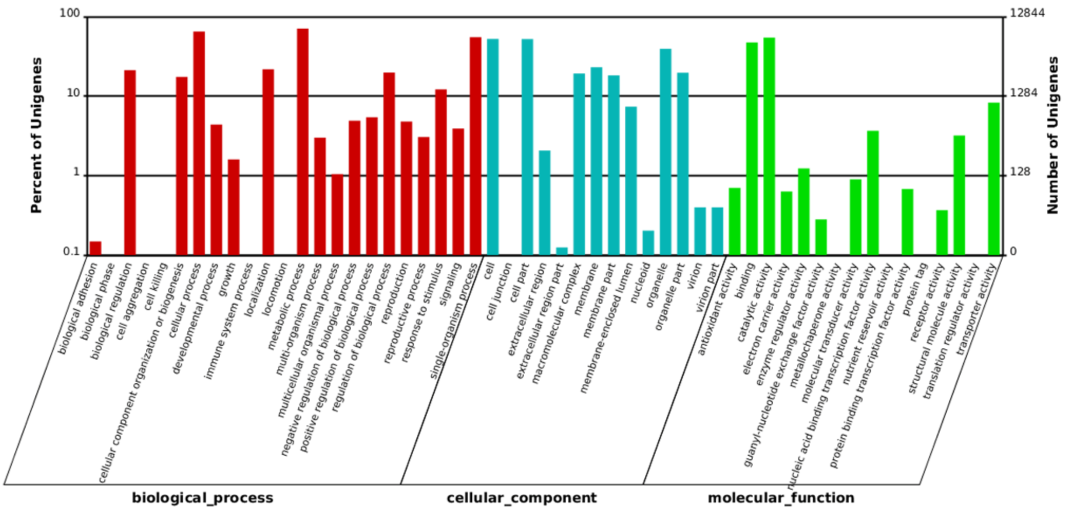

Figure S3. Gene Ontology (GO) classification of all unigenes. All unigenes were classified with respect to biological processes, molecular functions and cellular components.

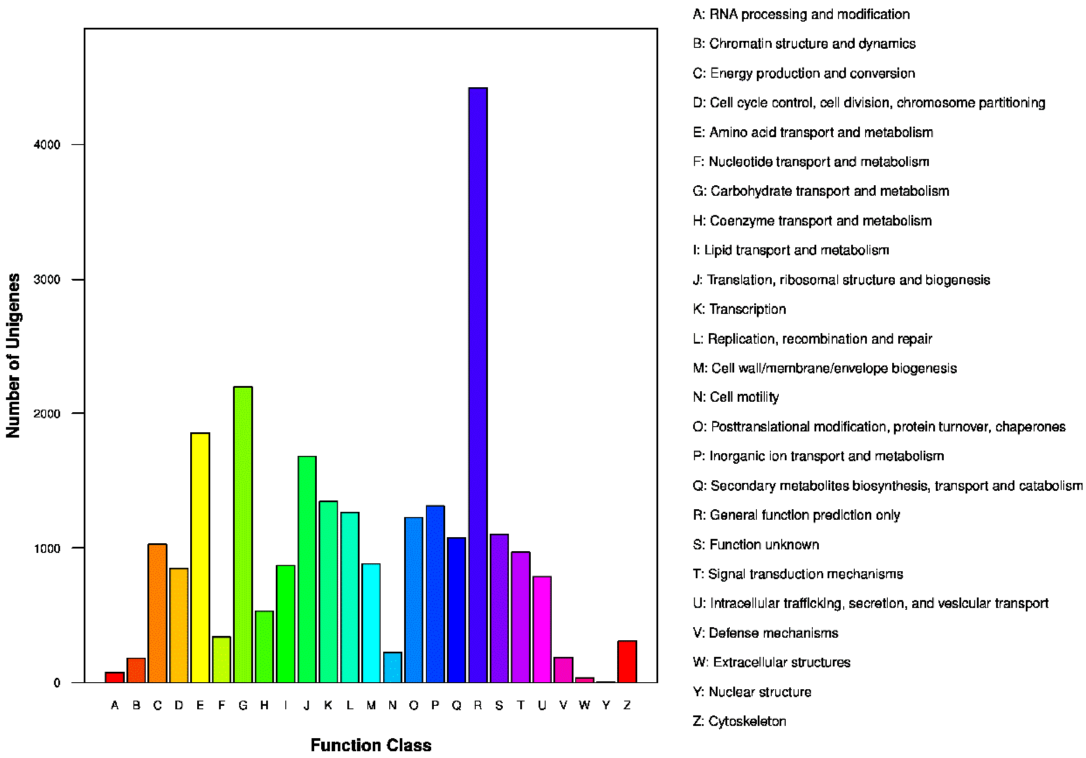

Figure S4. COG function classification of all unigenes.

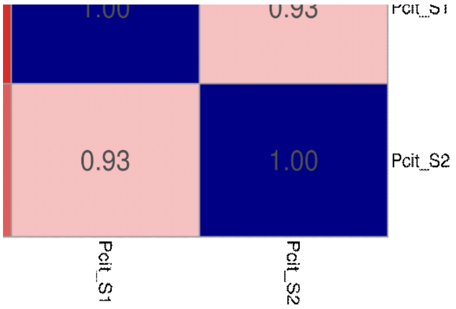

Figure S5. Spearman correlation analysis of the two replicates in each group. The vertical and horizontal axes represent the expression levels of all unigenes (log10 (FPKM)).

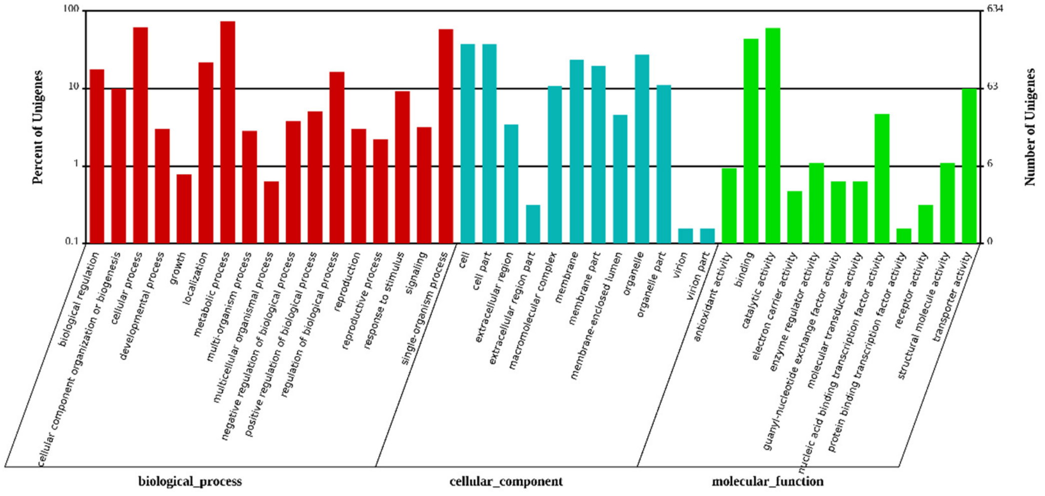

Figure S6. Cluster analysis of DEGs according to GO analysis. A total of 1085 DEGs were clustered using Blast2GO, and the analysis was performed with respect to biological processes, molecular functions and cellular components.

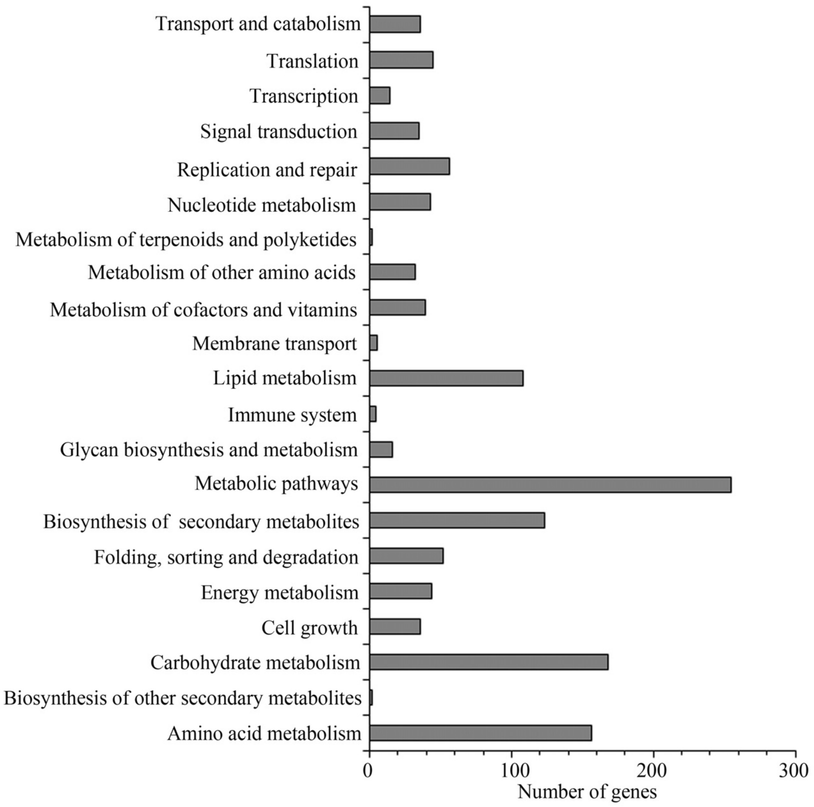

Figure S7. Cluster analysis of DEGs according to KEGG pathways. All those genes were classified according to the KEGG pathway database. Clusters of interest were selected for subsequent analysis.

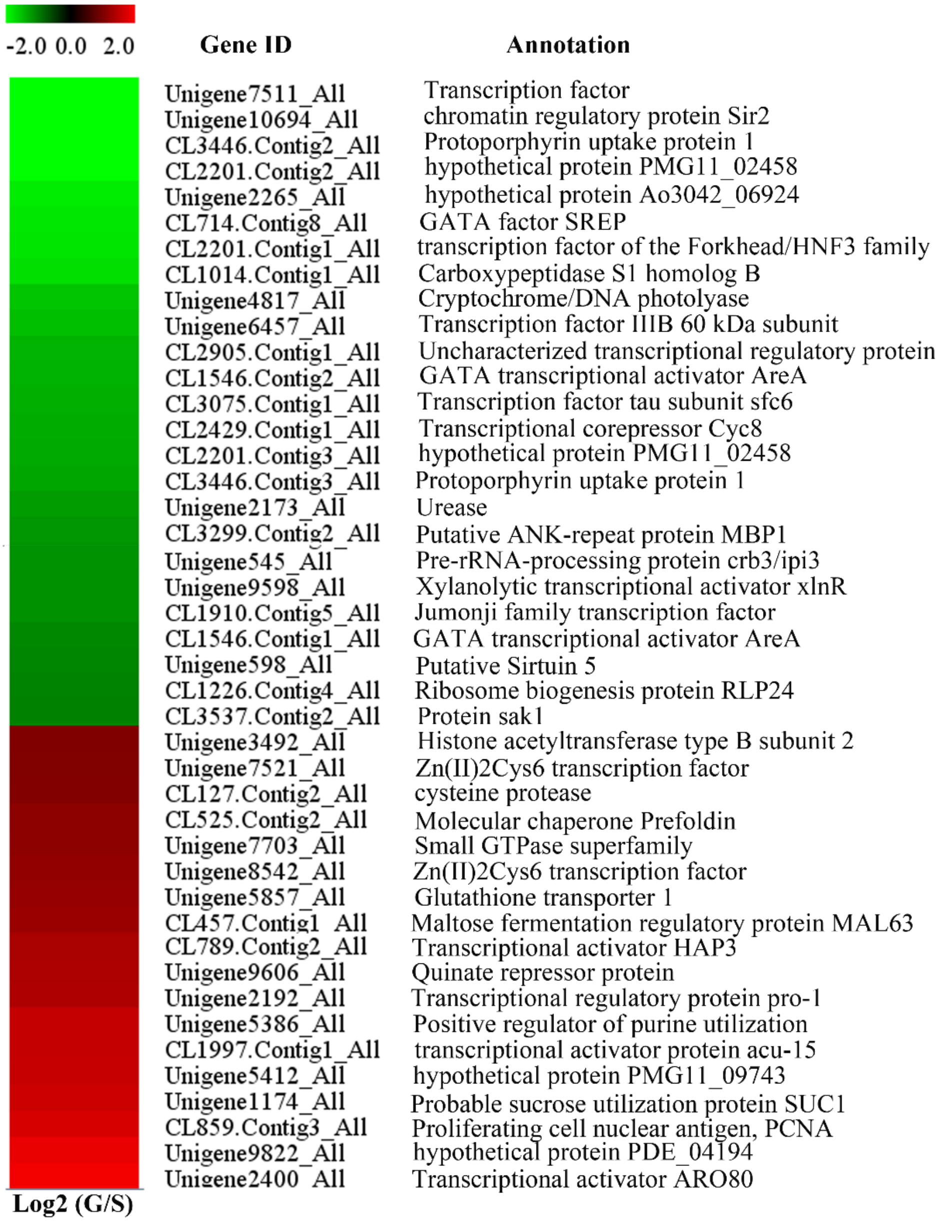

Figure S8. Expression profiles of DEGs involved in transcription factors.

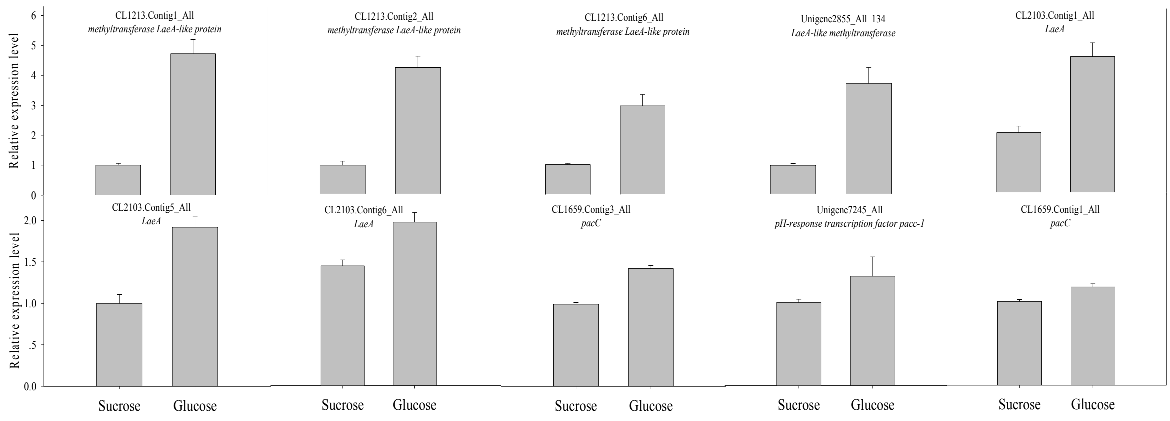

Figure S9. qRT-PCR analysis of expression levels of *LaeA* and *pacC* genes.

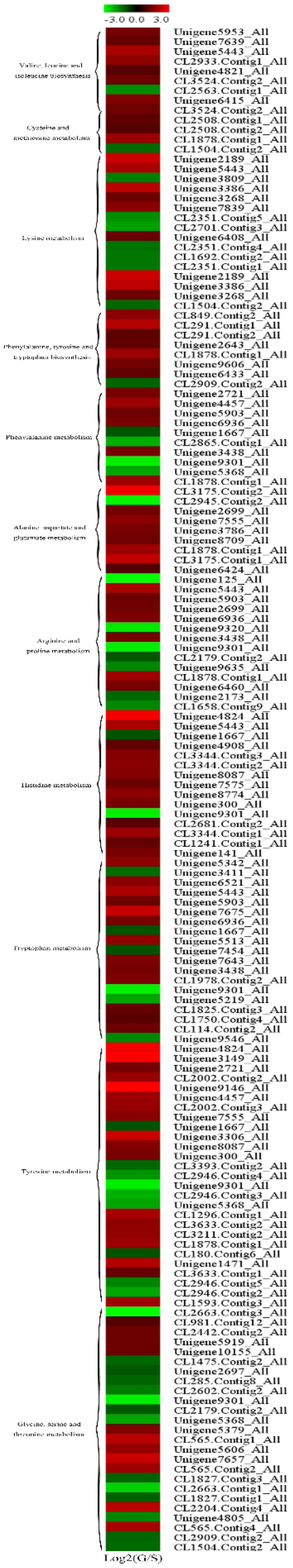

Figure S10. Expression profiles of DEGs involved in amino acids metabolism.
